# High‐fat diet effects on contractile performance of isolated mouse soleus and extensor digitorum longus when supplemented with high dose vitamin D

**DOI:** 10.1113/EP091493

**Published:** 2023-11-20

**Authors:** Sharn P. Shelley, Rob S. James, Steven J. Eustace, Emma L. J. Eyre, Jason Tallis

**Affiliations:** ^1^ Research Centre for Physical Activity, Sport and Exercise Science Coventry University Coventry UK; ^2^ Faculty of Life Sciences University of Bradford Bradford UK; ^3^ School of Life Sciences Coventry University Coventry UK

**Keywords:** fatigue, force, high‐fat diet, muscle function, obesity, power output, work loop

## Abstract

Evidence suggests vitamin D_3_ (VD) supplementation can reduce accumulation of adipose tissue and inflammation and promote myogenesis in obese individuals, and thus could mitigate obesity‐induced reductions in skeletal muscle (SkM) contractility. However, this is yet to be directly investigated. This study, using the work‐loop technique, examined effects of VD (cholecalciferol) supplementation on isolated SkM contractility. Female mice (*n =* 37) consumed standard low‐fat diet (SLD) or high‐fat diet (HFD), with or without VD (20,000 IU/kg^−1^) for 12 weeks. Soleus and EDL (*n =* 8–10 per muscle per group) were isolated and absolute and normalized (to muscle size and body mass) isometric force and power output (PO) were measured, and fatigue resistance determined. Absolute and normalized isometric force and PO of soleus were unaffected by diet (*P* > 0.087). However, PO normalized to body mass was reduced in HFD groups (*P* < 0.001). Isometric force of extensor digitorum longus (EDL) was unaffected by diet (*P* > 0.588). HFD reduced EDL isometric stress (*P* = 0.048) and absolute and normalized PO (*P* < 0.031), but there was no effect of VD (*P* > 0.493). Cumulative work during fatiguing contractions was lower in HFD groups (*P* < 0.043), but rate of fatigue was unaffected (*P* > 0.060). This study uniquely demonstrated that high‐dose VD had limited effects on SkM contractility and did not offset demonstrated adverse effects of HFD. However, small and moderate effect sizes suggest improvement in EDL muscle performance and animal morphology in HFD VD groups. Given effect sizes observed, coupled with proposed inverted U‐shaped dose‐effect curve, future investigations are needed to determine dose/duration specific responses to VD, which may culminate in improved function of HFD SkM.

## Abstract (highlights)

- **What is the central question of this study?**
  Can vitamin D supplementation alleviate detrimental effects of high‐fat diet (HFD) consumption on contractile performance of isolated skeletal muscles?
- **What is the main finding and its importance?**
  The present study is the first to examine the synergistic effects of HFD consumption and vitamin D supplementation on the contractile performance of isolated skeletal muscle. These findings suggest high dose vitamin D has limited effects on force, power or fatigue resistance of isolated mouse soleus and extensor digitorum longus.

## 1 INTRODUCTION

Obesity has quickly become a public health crisis, due to its links with poor physical (Chu et al., 2018) and mental well‐being (Talen & Mann, 2009), and increased risk of comorbidities and mortality (Abdelaal et al., 2017; Chu et al., 2018; Silvestris et al., 2018). Adequate skeletal muscle (SkM) function is key to maintaining whole‐body health (Frontera & Ochala, 2015); reductions in SkM function are associated with increased risk of non‐communicable disease, mortality and reduced quality of life (Murphy & Hartley, 2018; Wu et al., 2017). Adipose tissue, a connective tissue primarily composed of adipocytes, plays an important role in whole‐body homeostasis (Mittal, 2019). However, when there is a chronic positive energy balance, common in obese individuals, dysregulation of adipose tissue occurs leading to excess subcutaneous, visceral and ectopic accumulation (Longo et al., 2019). Excess adipose and ectopic fat impairs SkM metabolism (i.e., reduces lipid oxidation efficiency promoting further ectopic lipid accumulation; Mengeste et al., 2021), myogenesis and contraction resulting in a negative cycle of lipid accumulation (Tallis et al., 2018). Such reductions in SkM health associated with excess adipose exacerbate the adverse effects of obesity on whole‐body function and health.

Understanding of obesity's effects on SkM function has been advanced by high‐fat diet (HFD) and isolated skeletal muscle rodent models (Tallis et al., 2018). Such methods have distinct advantages over whole‐body in vivo assessments of muscle function, and have been outlined in recent reviews (Tallis et al., 2015, 2018, 2021). In brief, isolated skeletal muscle models allow for examination of direct muscle and fibre type‐specific effects and for control of dietary composition and duration, which are difficult to ascertain and control in human populations. Furthermore, isolated skeletal muscle models allow a more accurate evaluation of the direct impact of HFD on muscle quality (force normalized to cross‐sectional area or power normalized to muscle mass) given that the size of the contractile tissue can be more accurately determined, when compared to in vivo assessments. Understanding muscle quality is important as it gives an indication of HFD effects on the intrinsic force‐producing capacity of muscle (Tallis et al., 2018). Reductions in muscle quality are impactful in obese individuals, where in addition to an elevated cost of tissue failure, larger muscles are often formed, contributing to an already elevated bodily inertia (Tallis et al., 2017). In addition, direct examination of the effects of HFD on muscular fatigue is important for establishing if there is a reduction in the ability to sustain repetitive muscular contractions, opposed to an increase in muscle fatigability solely occurring in obese individuals due to the increase in force requirement of moving a larger mass.

Although effects of HFD consumption on isolated muscle function are contractile mode‐ and muscle‐specific, evidence suggests that HFD consumption can lead to a reduction in force and power normalized to body mass and muscle size (Tallis et al., 2018, 2021) and fatigue resistance (Hill et al., 2019; Hurst et al., 2019; Tallis et al., 2017). The prevalence and magnitude of HFD effects appear to be affected by HFD dietary composition, feeding duration, test temperature, age and sex (Tallis et al., 2018). Whilst few studies have directly examined both contractility and underpinning mechanisms associated with change in function, HFD‐induced reductions in contractile function have been attributed to excessive adiposity and intramuscular lipids (Messa et al., 2020), decreased AMP‐activated protein kinase (AMPK) activity (Tallis et al., 2017), impaired mitochondrial function (Heo et al., 2017), chronic inflammation (Erskine et al., 2017), impaired excitation contractile coupling (Eshima et al., 2020) and impaired myogenesis (D'Souza et al., 2015). Given the detrimental effects of HFD‐induced obesity on SkM health, and the link between SkM and whole‐body health, understanding strategies to ameliorate HFD effects on SkM function is important for the prevention and management of obesity and its associated comorbidities.

Calorific restriction and increased physical activity are well‐established lifestyle interventions for obesity management (Foster‐Schubert et al., 2012; Julian et al., 2018; Ross, 2000; Touati et al., 2011). However, adherence to such strategies is poor, attributed to requiring large lifestyle modification, which appears unsustainable for most. Nutritional supplements could be an appealing alternative to many weight management lifestyle interventions (i.e., increase physical activity or altered dietary intake), where the actual and perceived financial, time and physical burden are regularly cited as barriers to sustained behaviour change (Baillot et al., 2021; Darmon & Drewnowski, 2015). One such supplement is the pro‐hormone vitamin D_3_ (VD), which has been suggested as a potential nutritional strategy to reduce the impact of HFD consumption on SkM contractility (Tallis et al., 2021). VD deficiency (in the UK defined as serum 25‐hydroxyvitamin D (25(OH)D) concentrations <25 nmol/l; Lin et al., 2021) independent of obesity, is associated with impaired SkM function (Montenegro et al., 2019). An increasing body of evidence supports the notion that obesity may be a contributing factor leading to VD deficiency (VDD) (Duan et al., 2020); data suggest the prevalence of VDD is 35% higher in individuals with obesity compared to healthy‐weight counterparts (Pereira‐Santos et al., 2015). As such, VDD itself could be one of the primary mechanisms contributing to skeletal muscle dysfunction in obese individuals. Over recent years, chronic supplementation of VD has been increasingly explored in HFD rodent models (Alkharfy et al., 2012; Benetti et al., 2018; Fan et al., 2016; Marcotorchino et al., 2014; Montenegro et al., 2021; Trovato et al., 2018). There is evidence to suggest VD supplementation can reduce and attenuate the increase in both body mass and adiposity associated with HFD consumption (Benetti et al., 2018; Fan et al., 2016; Marcotorchino et al., 2014) through the inhibition of adipogenesis and increase in UCP3 activation (Fan et al., 2016). Furthermore, research indicates VD supplementation may ameliorate mechanisms reported to contribute to obesity‐induced declines in muscle function, such as modulating inflammation (Farhangi et al., 2017; Szymczak‐Pajor & Śliwińska, 2019) and promoting myogenesis (Cipriani et al., 2014), although the latter has not been directly examined in an obese animal model. Despite this mechanistic potential, to date there are no research studies which have examined the potential of VD for alleviating the decline in isolated muscle contractile function associated with HFD consumption.

Using previously established protocols for examining the impact of HFD on contractile performance of SkM (Hill et al., 2019; Hurst et al., 2019; Tallis et al., 2017), the present study uniquely examined the effects of 12 weeks of HFD, with and without high dose VD, on the maximal isometric force, work loop power output (PO) and fatigue resistance of soleus (predominately slow‐twitch) and extensor digitorum longus (EDL; predominantly fast‐twitch) muscle, isolated from young adult female CD‐1 mice.

## 2 METHODS

### 2.1 Ethical approval

Institutional (P108131) and UK Home Office (PP4247175) approval were granted for the use of animals and the procedures outlined in this study. All procedures were completed in accordance with the UK Animals (Scientific Procedures) Act 1986. All investigators understand the ethical principles under which the journal operates and certify that the present study complies with their animal ethics checklist.

### 2.2 Animals

**FIGURE 1:**
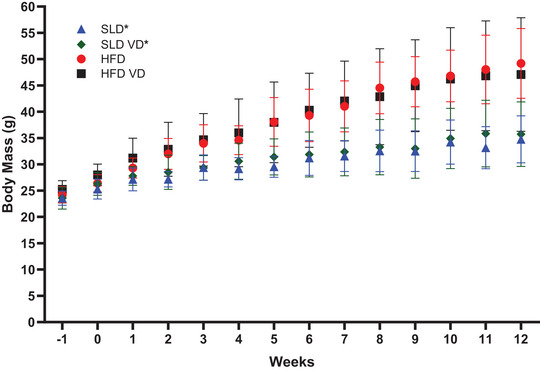
Effect of high‐fat diet and vitamin D on body mass over 12 weeks. Data presented as means ± SD; SLD, HFD, HFD VD, *n =* 9; SLD VD, *n =* 10. *Significant difference between HFD‐treated and SLD‐treated at *P* < 0.05; week −1 and 0 reflect habituation period where animals were gradually transitioned to their respective custom diet.

Female CD‐1 mice, purchased at ∼4 weeks old (Charles River, Margate, UK), were housed in groups of five at the University of Warwick and kept in 12:12 h light–dark cycles at 50% relative humidity, at 22°C. Mice were provided with ad libitum access to food and water. At 4 weeks of age, mice were randomly split into four experimental groups: standard low‐fat diet (SLD), HFD, SLD VD and HFD VD (total starting sample: *n* = 10 per group; final sample *n* = 10 SLD VD; *n =* 9 SLD, HFD and HFD VD). Following 13 days of habituation, which included gradual transition from standard lab chow (TestDiet 5755; calories provided by protein 18.3%, fat 22.1% and carbohydrate 59.6%; gross energy 4.07 kcal/g; VD (vitamin D_3_; cholecalciferol) 2200 IU/g) to new respective custom diets, mice consumed one of the following for 12 weeks: (1) standard low‐fat diet (TestDiet 58Y2; calories provided by protein 18.0%, fat 10.2% and carbohydrate 71.8%; gross energy 3.76 kcal/g; VD 900 IU/kg) (SLD); (2) high‐fat diet (TestDiet 58V8; calories provided by protein 18.1%, fat 46.2% and carbohydrate 35.7%; gross energy 4.62 kcal/g; VD 1200 IU/kg) (HFD); (3) SLD enriched with VD (20,000 IU/kg feed); and (4) HFD enriched with VD – as shown in Table 1. Animal health was monitored weekly using an Animal Welfare and Ethical Review Body (AWERB) approved checklist, which included monitoring body mass weekly (presented in Figure 1). Procedures were carried out blinded.

**TABLE 1:** Nutritional value of the diets given per experimental group.

Group	Standard low‐fat diet (TestDiet 58Y2) (SLD)	High‐fat diet (TestDiet 58V8) (HFD)	Standard low‐fat diet + vitamin D (SLD VD)	High‐fat diet + vitamin D (HFD VD)
Calories provided by				
Protein (%)	18.0	18.1	18.0	18.1
Fat (%)	10.2	46.2	10.2	46.2
Carbohydrates (%)	71.8	35.7	71.8	35.7
Energy (kcal g^−1^)	3.76	4.62	3.76	4.62
Fatty acid content				
Saturated (%)	1.14	9.05	1.14	9.05
Monounsaturated (%)	1.30	9.32	1.30	9.32
Polyunsaturated (%)	1.59	3.83	1.59	3.83
Vitamin D content (IU kg^−1^)	900	1200	20,000	20,000

Twelve weeks feeding duration and or 45% HFD was selected based on previous work that demonstrated large and statistically significant changes in body composition and muscle mechanics over similar durations (Bott et al., 2017; Eshima et al., 2020, 2017; Hurst et al., 2019; Matsakas et al., 2015). Furthermore, typical human Western diets may consist of ∼30–40% fat content, making a tolerable human HFD around 50–60%; fat content in a typical rodent diet is ∼10%, and thus 45% rodent HFD represents a similar dietary distortion to those found in standard and high‐fat human diets (Speakman, 2019). The dose of VD used (20,000 IU/kg^−1^ diet) was selected based on previous evidence indicating that similar high doses attenuate the increase in overall and segmental adipose tissue associated with HFD consumption over a 10 week feeding duration (15,000 IU/kg^−1^ diet) (Marcotorchino et al., 2014), and increase levels of serum 25(OH)D in blood plasma in HFD‐ and SLD‐treated mice (25,000 IU/kg^−1^ diet) (Park et al., 2020). Furthermore, an identical dose (20,000 IU/kg^−1^ diet) has previously been shown to elicit improvements in isolated skeletal muscle contractility in healthy mice, when compared to an apparently healthy, VD sufficient control group (Debruin et al., 2019; Hayes et al., 2019). Previous work indicates that 20,000 IU/kg^−1^ diet of VD equates to a daily intake of approximately 2,700 IU/kg^−1^ body mass in mice (Hayes et al., 2019). Following a previously determined calculation for converting dose in mice to dose in humans (daily animal dose per kg body mass ÷ metabolic scaling factor for mice; Nair & Jacob, 2016), the human equivalent dose of vitamin D is ∼13,000IU^−1^ day for a 60 kg individual.

### 2.3 Muscle preparations

Following the 12‐week treatment period, animals were culled via cervical dislocation (in accordance with the UK Animals (Scientific Procedures) Act 1986, Schedule 1). Animals were then weighed, to determine body mass (BM), and nasoanal length (NAL) was measured using digital callipers (Fisher Scientific 3417, Fisher Scientific, Loughborough, UK). From this information body mass index (BMI; Sjögren et al., 2001) and Lee index of obesity (Bernardis & Patterson, 1968) were calculated for each mouse.

(1) $$\mathrm{Body}\;\mathrm{mass}\;\mathrm{index}=\frac{\mathrm{BM}\left[g\right]}{\left(\mathrm{NAL}{\left[\mathrm{cm}\right]}^{2}\right)}/100$$

(2) $$\mathrm{Lee}\;\mathrm{Index}\;\mathrm{of}\;\mathrm{Obesity}=\frac{\sqrt[{3}]{\mathrm{BM}\left[g\right]}}{\mathrm{NAL}\left[\mathrm{cm}\right]}\times 1000$$

The subcutaneous fat pad around the top of the hindlimbs and genitals was extracted and weighed as an indication of overall adiposity (Rogers & Webb, 1980) and was later normalized to body mass for a measure of relative adiposity (Hill et al., 2019). In addition, whole extensor digitorum longus (EDL; *n =* 10 for SLD VD, *n =* 9 for HFD and HFD VD, *n =* 8 for SLD) from the right hind limb and soleus (*n =* 10 for SLD VD, *n =* 9 for HFD VD and *n =* 8 for SLD and HFD) from the left hindlimb were dissected in refrigerated (1–3°C) oxygenated (95% O_2_, 5% CO_2_) Krebs–Henseleit solution (in mM: NaCl 118; KCl 4.75; MgSO_4_ 1.18; NaHCO_3_ 24.8; KH_2_PO_4_ 1.18; glucose 10; CaCl_2_ 2.54 ; pH 7.55 at room temperature before oxygenation). To avoid pseudoreplication only one EDL and soleus was isolated per animal. The soleus and EDL represent locomotor muscles which differ in fibre type and function (soleus 53.6% type I, 31.2% type IIA, 15.2% type IIX; EDL 3.9% type I; 9.3% type IIX; 86.8% type IIB) (Agbulut et al., 2003), which allow for a greater understanding of any potential muscle and fibre type‐specific effects of HFD and VD. For each muscle preparation, the tendon attachment at the proximal end was left intact with a small piece of bone still attached, and aluminium foil T‐clips were wrapped around the distal tendon(s) as close to the muscle as possible to avoid slippage when the muscle was producing force (Ford et al., 1977; Goldman & Simmons, 1984; James et al., 2005).

### 2.4 Contractility measures

Upon dissection the muscle was placed in a Perspex flow‐through chamber filled with circulated constantly oxygenated (95% O_2_, 5% CO_2_) Krebs–Henseleit solution. The reservoirs of Krebs solution were stored in external heater/cooler baths (Grant LTD6G, Grant Instruments, Shepreth, UK), which were adjusted to maintain a physiologically relevant temperature of 37 ± 0.2°C inside the muscle bath. Temperature in the bath was continuously monitored using a digital thermometer (Fisher Traceable, Fisher Scientific). Using the bone at the proximal end, the muscle was attached to a crocodile clip connected to a force transducer (UF1, Pioden Controls Ltd, Henwood/Ashford, UK) and the T‐foil clip at the distal end was attached to a crocodile clip, connected to a motor arm (V201, Ling Dynamic Systems, Royston, UK).

### 2.5 Isometric force

The procedures utilized to measure the contractile properties of isolated mouse EDL and soleus in this study are based on well‐established protocols. For the assessment of isometric contractile properties, platinum electrodes running parallel to the muscle within the bath received an electrical stimulation from a tabletop power supply to activate the muscle (PL320, Thurlby Thandar Instruments, Huntington, UK). Stimulation parameters were controlled using custom‐written software (Testpoint, CEC, Norton, USA), via a D/A board (KPCI3108, Keithley Instruments, Cleveland, USA), on a standard desktop personal computer. Muscle length and stimulation amplitude (typically 12–16 V for SOL and 14–18 V for EDL) were optimized to elicit a maximal isometric twitch response, the magnitude of which was determined via a digital storage oscilloscope (2211 or 1002, Tektronix, Marlow, UK). Using the optimized muscle length and stimulation amplitude, stimulation frequency was manipulated (100–130 Hz (typically 120 Hz) and 200–230 Hz (typically 230 Hz) for the SOL and EDL, respectively) to elicit a maximal isometric tetanus, with a 5‐min rest implemented between each tetanus (Askew & Marsh, 1997; Shelley et al., 2022; Vassilakos et al., 2009). In all cases, burst duration was fixed at 350 ms for SOL and 250 ms for EDL. After maximal force had been achieved a control tetanus was performed at the first stimulation frequency to monitor change in contractile performance over time. From the maximal tetanus response, time to half peak tetanus force (TTHP) and last stimulus to half relaxation (LSHR) were recorded as indicative measures of activation and relaxation kinetics, respectively (Ebashi & Endo, 1968). The muscle length optimal for isometric performance, defined as *L*_0_, was measured using an eyepiece graticule and microscope. Estimated fibre length for the SOL and EDL were calculated as 85% and 75% of *L*_0_, respectively (James et al., 1995).

### 2.6 Work‐loop power output

The work‐loop (WL) technique, which has been used in previous research examining the effects of HFD on mouse SkM function (Hill et al., 2019; Hurst et al., 2019; Tallis et al., 2017), was utilized to examine the contractile performance of isolated muscle during cyclical length changes, which better replicates in vivo dynamic muscle activity when compared to other in vitro assessments (James et al., 1995, 1996; Josephson, 1985, 1993). The WL technique involved each muscle being subjected to four sinusoidal length changes around the previously determined *L*_0_. During the length change cycle, muscle was stimulated to produce force primarily during the shortening phase, using the previously determined stimulation amplitude. Stimulation frequency was increased from that which resulted in maximal isometric performance to 160 and 260 Hz for the SOL and EDL, respectively, as previous work indicates that a greater stimulation frequency is required for maximal WL PO (Shelley et al., 2022; Vassilakos et al., 2009). Length changes were performed via movement of the motor arm (V201, Ling 220 Dynamic Systems) controlled via a custom written computer program (Testpoint, CEC), the position of which was measured using a Linear Variable Displacement Transformer (DFG5.0, Solartron Metrology, Bognor Regis, UK). During the length change cycle, instantaneous force and velocity were sampled at 10 kHz and plotted against each other, forming a WL. The instantaneous power (instantaneous velocity × instantaneous force) calculated at each time point during the WL was averaged over the entire WL to determine net power output. A range of cycle frequencies (CF; rate at which each muscle undergoes sinusoidal length change cycle) were utilized to examine whether the CF that elicited maximal PO was influenced by a HFD or VD.

Initially, CFs of 5 and 10 Hz, burst durations of 65 and 50 ms, phase shift of −10 ms and −2 ms (time of initiation of stimulation prior to muscle reaching its greatest length), and a strain of 0.10 (muscle lengthening by 5%, shortening by 10% and elongating by 5% back to *L*_0_) were used to examine the SOL and EDL, respectively, as previous work has established that these muscles typically achieve maximal WL PO using these parameters (James et al., 1995; Tallis et al., 2017). Burst duration, phase and strain were all subject to change based on WL shape to maximize net work, for example, where WL shape sloped downwards during re‐lengthening, as the muscle was still undergoing relaxation, burst duration was reduced to achieve maximal WL PO. Every 5 min, a set of four WLs were performed, and maximal net work was recorded and later used to quantify power production. Once parameters for optimal PO at 5 and 10 Hz CF for the soleus and EDL, respectively, were identified, these CFs were then used as the control WLs for the remainder of the experiment. Once maximal WL PO had been achieved at the control CFs, power output was then assessed across a range of CFs to establish a PO–CF curve for each experimental group (Hill et al., 2019, 2020; Hurst et al., 2019). The range of CFs used, and typical parameters utilized to elicit maximal net work of each muscle at each CF, are displayed in Table 2, with the exception of phase, which was typically −10 ms for the SOL and −2 ms for the EDL; at higher CFs phase would occasionally be reduced to ensure stimulation occurred at the onset of shortening, for example, at 10 Hz for the soleus −6 ms phase was often implemented. On rare occasions strain would be increased beyond that provided in Table 2 (0.2 increments) at lower CFs when WLs appeared rectangular in shape, again to ensure maximal net work. Except for the control CFs, the order of CFs was randomized using Microsoft Excel (Windows v. 2016). After every three CFs, and after the final CF examined, a control set of WLs were performed using the initial parameters to examine the deterioration of power over time as the performance of isolated mouse muscle will deteriorate over time due to the build‐up of an anoxic core (Barclay, 2005). Utilising this approach allowed for the correction of net work for other CFs relative to the control WLs, as has been common practice in previous research (Hill et al., 2020; Hurst et al., 2019; Vassilakos et al., 2009). Once the final control WL was performed, the muscle rested for 10 min prior to assessment of fatigue and recovery.

**TABLE 2:** Typical stimulus burst durations and strains that elicited maximal net work, at each cycle frequency, during the WL assessment of soleus and EDL.

		Cycle Frequency (Hz)										
Muscle	Parameter	2	3	4	5	6	7	8	10	12	14	16
Soleus	Burst Duration (ms)	245	150	92	65	52	35	24	11	—	—	—
	Strain	0.13	0.12	0.11	0.1	0.09	0.08	0.07	0.06	—	—	—
EDL	Burst Duration (ms)	—	—	110	—	75	—	65	50	32	24	16
	Strain	—	—	0.13	—	0.12	—	0.11	0.1	0.09	0.08	0.07

*Note*: Dash (—) represents CFs not used for assessment of contractile performance. Values provided were subject to change based on the shape of WL to ensure maximal WL PO.

### 2.7 Fatigue resistance, cumulative work and recovery

For examination of fatigue resistance, each muscle preparation was subjected to 50 consecutive WL cycles, using the parameters that elicited maximal WL PO at 5 and 10 Hz CF for the soleus and EDL, respectively. However, stimulation frequency was reduced to 100 and 200 Hz for the soleus and EDL, respectively, to better represent the in vivo mechanism of fatigue as suggested by previous work (Shelley et al., 2022; Vassilakos et al., 2009). The network of every loop was recorded and plotted relative to maximal PO recorded during the fatigue protocol. The time taken for power to fall below 50% maximal power output was recorded, as has been used in previous studies examining fatigue resistance and dietary‐induced obesity using isolated mouse muscle (Hill et al., 2019; Hurst et al., 2019; Tallis et al., 2017). Cumulative work, calculated as the sum of the net work performed in each cycle (Askew et al., 1997; Vassilakos et al., 2009) was also determined to infer absolute differences in the fatigue response, accounting for potential differences in absolute power output between experimental groups, which may promote a faster rate of fatigue. WL shapes were plotted as force against strain (%*L*_0_) for the individual force and length data points for each work loop cycle.

The ability of each muscle preparation to recover from the fatigue protocol was monitored every 10 min for 30 min post‐fatigue. Every 10 min the muscle was subjected to one set of WL cycles using the same contractile parameters for the control WLs. Net work during recovery was expressed as a percentage of pre‐fatigue maximal power output.

### 2.8 Muscle mass and dimensions calculations

Upon completion of contractile assessments, which from the point of animal death to final contractile assessment lasted ∼210 min, the muscle was detached from the crocodile clips and removed from the muscle chamber. The tendons, T‐foil clip and bone were removed leaving only muscle tissue, which was then blotted on absorbent paper to remove the excess Krebs solution and weighed to determine wet muscle mass (TL‐64, Denver Instrument Company, Arvada, CO, USA). Mean muscle cross‐sectional area was calculated from *L*_0_, muscle mass and an assumed density of 1060 kg m^−3^ (Méndez & Keys, 1960). Isometric stress (kN m^−2^) was calculated by dividing peak tetanic force by mean muscle cross sectional area (CSA). WL PO normalized to body mass (W kg^−1^ body mass) was calculated by dividing absolute PO by body mass. WL PO normalized to muscle mass (W kg^−1^ muscle mass) was calculated as an indicative measure of muscle quality.

### 2.9 Statistical analysis

Statistical analysis was performed using SPSS Statistics for Windows v.25 (IBM Corp, Armonk, NY, USA) and GraphPad Prism v.9 (GraphPad Software, Boston, MA, USA). All data are presented as means ± standard deviation (SD). Following appropriate checks of normality (Shapiro–Wilk) and homogeneity of variance (Levene's test), parametric analysis was performed. Mixed model analysis of variance (ANOVA), with HFD, treatment (between subjects) and weeks (within subjects) used as the factors, was utilized to determine the effect of HFD and VD consumption on weekly body mass. Two‐factor ANOVA was conducted with HFD (SLD vs. HFD) and treatment (Control vs. VD) as the fixed factors to examine if differences existed in measures of animal and muscle morphology (body mass, muscle mass, muscle length and muscle CSA), isometric properties (absolute tetanic force, tetanic stress, time to half peak tetanus (THPT) and LSHR), time to reach 50% of maximal PO during fatigue protocol and cumulative work produced after 50 consecutive WLs. Mixed model repeated measures ANOVA, with HFD, treatment (between subjects) and CF (within subjects) used as the factors, was utilized to determine any changes in SOL and EDL absolute PO and PO normalized to body mass and muscle mass. Mixed model repeated measures ANOVA, with HFD, treatment (between subjects) and stimulation frequency (within subjects) used as the factors, was utilized to determine if the reduction in stimulation frequency used for assessment of fatigue resulted in any changes in SOL and EDL PO normalized to muscle mass and if so, whether the changes in PO were uniform across diet and treatment groups. Mixed model repeated measures ANOVA, with HFD, treatment (between subjects) and time (within subjects) as the factors, was utilized to examine the recovery of WL PO following the fatigue protocol. Significant interactions observed for ANOVA were explored using the Bonferroni *post hoc* test for multiple comparisons. Partial eta square (η_p_^2^) was calculated to estimate effect sizes for all significant main effects. Thresholds for η_p_^2^ effect size were classified as small (<0.05), moderate (0.06–0.137) or large (>0.138) (Cohen, 1988). Where possible, Cohen's *d* was calculated to measure effect size of interactions observed and was then corrected for bias using Hedge's *g* due to differences in sample size between groups (Hedges, 1981). Hedge's *g* effect size was interpreted as trivial (<0.2), small (0.2–0.6), moderate (0.6–1.2) or large (>1.2) (Hopkins et al., 2009). The level of significance was set at *P* ≤ 0.05.

SPM‐1D package (^©^Todd Pataky, version M 0.1) in MATLAB (R2018b, The MathWorks Inc, Natick, MA, USA) was used to perform statistical parametric mapping (SPM) on cumulative work and percentage decline in PO data (Pataky, 2010). SPM calculates the *F* (ANOVA) or *t* (Student's *t*‐test) value on every data point obtained during the fatigue protocol; instead of calculating a *P*‐value for every data point, inferential statistics are based on random field theory and thus maintain a constant error of α (Pataky et al., 2013). First, a one‐way ANOVA SPM[*F*] statistic was used to determine if there was a main effect of group (SLD, SLD VD, HFD, HFD VD) on cumulative work and percentage decline in fatigue relative to maximum PO. Where clusters crossed the critical threshold a *P‐*value was calculated (Pataky et al., 2013). Where main effects were observed, a *post hoc* two‐sample SPM[*t*] two‐sided *t*‐test was used between each group (SLD, SLD VD, HFD, HFD VD) separately to identify which groups differed and where the differences occurred (Pataky et al., 2015). For EDL cumulative work production SLD and SLD VD groups, and HFD and HFD VD groups did not differ, and as such they were pooled together to form a SLD versus HFD comparison. Where clusters crossed the critical threshold, this indicated a significant difference at *P* ≤ 0.05.

## 3 RESULTS

### 3.1 Morphology

There was a significant time × HFD interaction observed for weekly body mass (Figure 1. *P* < 0.001, η_p_^2^ = 0.412). Bonferroni *post hoc* analysis multiple comparisons revealed HFD‐treated animals had significantly greater body mass from week 1 onwards (*P* < 0.048). No effect of VD was observed (*P* > 0.999).

Final whole animal body mass, body length, BMI, Lee Index of Obesity, fat mass and adiposity:body mass were significantly greater in HFD groups compared to SLD groups (*P* < 0.005, η_p_^2^ > 0.213; Table 3). No significant differences were observed for EDL or soleus muscle mass, fibre length or CSA (*P* > 0.053, η_p_^2^ < 0.112; Table 3).

**TABLE 3:** Whole body and muscle specific animal morphology.

					HFD effect		VD effect		Interaction	
	SLD	SLD‐VD	HFD	HFD‐VD	*P*	η_p_^2^	*P*	η_p_^2^	*P*	η_p_^2^
Whole body	*n =* 9	*n* = 10	*n* = 9	*n* = 9						
Body mass (g)	35.0 ± 4.7	34.4 ± 6.6	48.8 ± 4.4	48.4 ± 7.9	**<0.001**	**0.566**	0.814	0.002	0.991	<0.001
Body length (mm)	101 ± 5	102 ± 5	106 ± 3	108 ± 6	**0.005**	**0.213**	0.313	0.031	0.964	<0.001
Fat mass (g)	1.1 ± 0.6	0.9 ± 0.9	4.6 ± 1.7	3.5 ± 1.4	**<0.001**	**0.627**	0.152	0.061	0.328	0.029
Lee Index of Obesity	325 ± 8.7	317 ± 12.7	346 ± 11.2	338 ± 12.7	**<0.001**	**0.458**	0.061	0.102	0.985	<0.001
BMI (g cm^−2^)	0.34 ± 0.02	0.33 ± 0.03	0.44 ± 0.04	0.42 ± 0.04	**<0.001**	**0.599**	0.149	0.062	0.889	<0.001
Adiposity:body mass	2.8 ± 1.4	2.2 ± 1.8	9.3 ± 3.2	7.1 ± 1.9	**<0.001**	**0.627**	0.063	0.101	0.313	0.031
EDL	*n* = 8	*n* = 10	*n* = 9	*n* = 9						
Muscle mass (mg)	10.7 ± 1.6	10.8 ± 0.9	11.3 ± 0.7	11.4 ± 0.8	0.053	0.112	0.65	0.007	0.993	<0.001
Fibre length (mm)	9.2 ± 0.3	9.1 ± 0.4	8.9 ± 0.5	9.2 ± 0.6	0.496	0.015	0.306	0.033	0.220	0.047
CSA (m^2^)	1.10 × 10^−6^ ± 1.26 × 10^−7^	1.11 × 10^−6^ ± 1.13 × 10^−7^	1.21 × 10^−6^ ± 8.97 × 10^−8^	1.18 × 10^−6^ ± 1.63 × 10^−7^	0.064	0.103	0.953	<0.001	0.608	0.008
SOL	*n* = 8	*n* = 10	*n* = 8	*n* = 9						
Muscle mass (mg)	8.7 ± 1.5	7.9 ± 1.0	8.4 ± 0.6	8.8 ± 0.8	0.422	0.021	0.457	0.012	0.130	0.072
Fibre length (mm)	9.4 ± 0.5	9.5 ± 0.6	9.5 ± 0.5	9.4 ± 0.4	0.940	<0.001	0.696	0.005	0.528	0.013
CSA (m^2^)	8.77 × 10^−7^ ± 1.37 × 10^−7^	7.82 × 10^−7^ ± 6.14 × 10^−8^	8.40 × 10^−7^ ± 9.32 × 10^−8^	8.78 × 10^−7^ ± 8.53 × 10^−8^	0.407	0.022	0.422	0.021	0.066	0.105

*Note*: Values are presented as means ± SD; bold values indicate a significant difference at *P* < 0.05. CSA: Cross‐sectional area.

### 3.2 Isometric performance

There were no significant effects of HFD or VD on soleus isometric properties (Table 4. *P* > 0.087, η_p_^2^ < 0.092) or EDL THPT and tetanus force (*P* > 0.385, η_p_^2^ < 0.024; Table 4). However, EDL from HFD groups produced significantly lower tetanus stress and slower LSHR (*P* < 0.048, η_p_^2^ > 0.117; Table 4) when compared to SLD groups.

**TABLE 4:** The effect of 12‐week high‐fat diet and vitamin D on the isometric properties of isolated mouse EDL and soleus.

					HFD effect		VD effect		Interaction	
	SLD	SLD‐VD	HFD	HFD‐VD	*P*	η_p_^2^	*P*	η_p_^2^	*P*	η_p_^2^
EDL	*n* = 8	*n* = 10	*n* = 9	*n* = 9						
Tetanus Force (mN)	364 ± 78	358 ± 49	340 ± 58	354 ± 90	0.588	0.009	0.869	0.001	0.699	0.005
Tetanus stress (kN m^−2^)	333 ± 64	320 ± 34	283 ± 52	297 ± 49	**0.048**	**0.117**	0.975	<0.001	0.470	0.016
THPT (ms)	11.4 ± 1.9	10.8 ± 2.4	10.4 ± 1.8	10.5 ± 1.9	0.385	0.024	0.748	0.003	0.614	0.008
LSHR (ms)	13.2 ± 1.1	13.4 ± 1.4	15 ± 1.3	14.5 ± 2.0	**0.013**	**0.178**	0.751	0.003	0.505	0.014
Soleus	*n* = 8	*n* = 10	*n* = 8	*n* = 9						
Tetanus force (mN)	236 ± 48	208 ± 32	203 ± 35	220 ± 22	0.436	0.020	0.658	0.006	0.084	0.093
Tetanus stress (kN m^−2^)	278 ± 50	266 ± 30	243 ± 40	252 ± 34	0.087	0.092	0.927	<0.001	0.428	0.019
THPT (ms)	34.5 ± 6.7	35.7 ± 5.1	36.4 ± 5.4	33.4 ± 3.8	0.935	<0.001	0.654	0.007	0.281	0.037
LSHR (ms)	40.1 ± 7.8	47.6 ± 8.3	45.9 ± 6.6	47.8 ± 7.8	0.292	0.036	0.100	0.085	0.321	0.032

*Note*: Values are presented as means ± SD; bold values indicate a significant difference at *P* < 0.05. LSHR, last stimulus half‐relaxation time; THPT, time to half peak tetanus.

### 3.3 Maximal work loop power output

**FIGURE 2:**
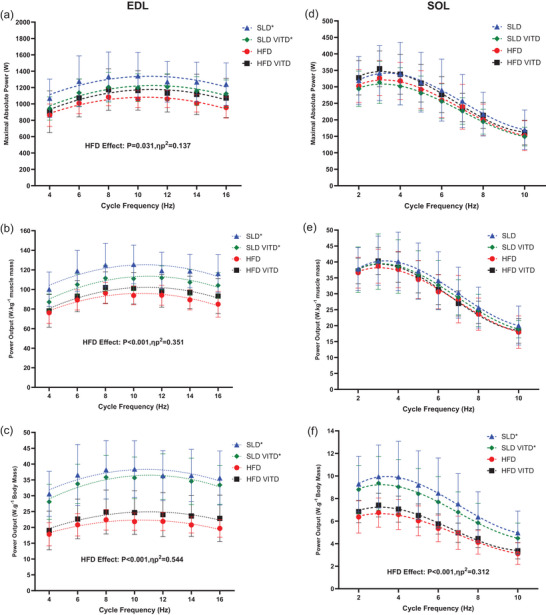
The effect of 12 weeks’ high‐fat diet and vitamin D on the power output–cycle frequency relationship for absolute power output (watts), power output normalized to muscle mass (watts per kg of muscle mass) and power output normalized to body mass (watts per gram of body mass) of isolated mouse EDL (a, b and c, respectively) and soleus (d, e and f, respectively). For EDL: *n =* 10 for SLD VD; *n =* 9 for HFD and HFD VD; *n =* 8 for SLD; for soleus: *n =* 10 for SLD VD; *n =* 9 for HFD VD; and *n =* 8 for SLD and HFD. Data presented as means ± SD. *Significant difference at *P* < 0.05.

For EDL, absolute PO and PO normalized to muscle mass, there was a significant main effect of CF (*P* < 0.001, η_p_^2^ > 0.696) indicating peak PO occurred between 8 and 12 Hz cycle frequency (Figure 2a,b; *P* > 0.264). For PO normalized to body mass there was a significant CF × HFD interaction (*P* = 0.018, η_p_^2^ = 0.092). Bonferroni multiple comparisons indicate that in the SLD groups peak PO occurred between 8 and 12 Hz (*P* > 0.999). In the HFD‐treated groups peak PO normalized to body mass occurred between 8 and 14 Hz (*P* > 0.073).

EDL absolute PO and PO normalized to muscle mass and body mass were significantly lower in the HFD groups when compared to SLD groups (Figure 2a,b,c; *P* < 0.031, η_p_^2^ > 0.137). No main effect of VD (*P* > 0.493, η_p_^2^ < 0.015) or HFD × VD interaction was observed (*P* > 0.070, η_p_^2^ < 0.099). There were no CF × HFD (*P* > 0.492, η_p_^2^ < 0.025), CF × VD (*P* > 0.409, η_p_^2^ < 0.030) or CF × HFD × VD (*P* > 0.303, η_p_^2^ < 0.037) interactions observed.

For soleus, absolute PO and PO normalized to muscle mass, there was a significant main effect of CF (*P* < 0.001, η_p_^2^ > 0.913), indicating maximal PO occurred between 3 and 4 Hz cycle frequency (*P* > 0.184). No CF × HFD (*P* < 0.372, η_p_^2^ < 0.032), CF × VD (*P* > 0.636, η_p_^2^ < 0.016) or CF × HFD × VD (*P* > 0.510, η_p_^2^ < 0.023) interaction was observed. For PO normalized to body mass there was a significant CF × HFD interaction (*P* = 0.012, η_p_^2^ = 0.128). However, Bonferroni multiple comparisons indicated that irrespective of group, maximal absolute power occurred between 3 and 4 Hz cycle frequency (*P* > 0.999).

For soleus, PO normalized to body mass, HFD‐treated groups produced significantly less power than SLD groups (Figure 2f; *P* = 0.001, η_p_^2^ = 0.312). For absolute PO and PO normalized to muscle mass, no main effects of HFD (*P* > 0.471, η_p_^2^ < 0.017) or VD (*P* > 0.722, η_p_^2^ < 0.004) were observed. No HFD × VD interaction was observed (*P* > 0.250, η_p_^2^ < 0.042) for absolute or normalized PO.

### 3.4 Fatigue and recovery

**FIGURE 3:**
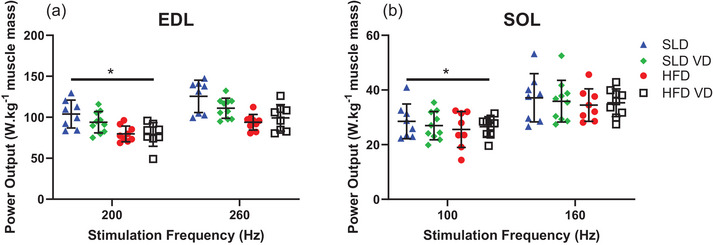
The effect of stimulation frequency and 12 weeks’ high‐fat diet and vitamin D on maximal work loop power output normalized to muscle mass (watts per kg of muscle mass) of isolated mouse EDL (a) and soleus (b). For EDL: *n =* 10 for SLD VD; *n =* 9 for HFD; *n =* 8 for SLD and HFD VD; for soleus: *n =* 10 for SLD VD; *n =* 9 HFD VD; *n =* 8 for SLD and HFD. Data presented as means ± SD. *Significant difference between stimulation frequencies at *P* < 0.05.

The reduction in stimulation frequency for assessment of fatigue resulted in a significant reduction in maximal WL PO normalized to muscle mass of the soleus and EDL (Figure 3; *P* < 0.001, η_p_^2^ > 0.725), but the magnitude of reduction in PO was not influenced by diet or treatment (*P* > 0.199, η_p_^2^ < 0.053).

**FIGURE 4:**
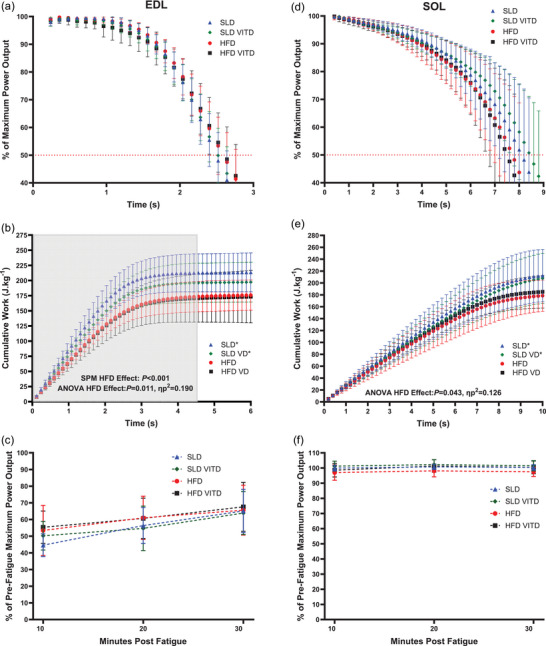
The effect of 12 weeks’ high‐fat diet and vitamin D on net muscle power output relative to maximum during fatigue (a, d), on cumulative work normalized to muscle mass (joules per kg muscle mass) produced during 50 consecutive work‐loop cycles (b, e), and on the recovery of maximal work loop power output over 30 min post‐fatigue (c, f) of isolated mouse EDL (a, b, c) and soleus (d, e, f). Shaded area of (b) indicates significant difference between SLD and HFD groups determined via two‐sample SPM[*t*] statistical analysis. For EDL *n =* 10 for SLD VD, *n =* 9 for HFD, *n =* 8 for SLD and HFD VD; for soleus *n =* 10 for SLD VD; *n =* 9 HFD VD and *n =* 8 for SLD and HFD. Data presented as means ± SD. *Significant difference between SLD and HFD‐treated groups at *P* < 0.05.

For time to fall below 50% of maximum power output during fatigue, there were no significant effects of HFD (Figure 4a, d; *P* > 0.220, η_p_^2^ < 0.048), VD (*P* > 0.962, η_p_^2^ < 0.001) or HFD × VD interactions observed (*P* > 0.455, η_p_^2^ < 0.018) in either the EDL or soleus. Statistical parametric mapping (SPM) ANOVA[*F*] also indicated no significant differences between groups for percentage decline of PO relative to maximum (Figure 4a, d, *P >* 0.999).

HFD groups produced significantly less cumulative work after 50 consecutive WLs when compared to SLD groups in both the EDL (SLD, 213.9 ± 9.5; SLD VD, 197.2 ± 9.8; HFD, 175.9 ± 7.5; HFD VD, 173.4 ± 12.8 J kg^−1^) and soleus (SLD, 212.5 ± 13.0; SLD VD, 207.8 ± 12.6; HFD, 178.6 ± 7.7; HFD VD, 185.1 ± 8.2 J kg^−1^; Figure 4b, e; *P* < 0.043, η_p_^2^ > 0.126). There was no significant main effect of VD (*P* > 0.417, η_p_^2^ < 0.021) or HFD × VD interaction observed (*P* > 0.545, η_p_^2^ < 0.012). SPM ANOVA[*F*] also indicated a significant effect of experimental group on cumulative work production for the EDL (Figure 4b, *P* < 0.001) but not for the soleus (Figure 4e, *P >* 0.999). The SPM[*t*] *t*‐test results for EDL cumulative work indicated no differences between SLD and SLD VD groups or HFD and HFD VD groups indicating no effect of VD, as such groups were pooled together to form a SLD vs. HFD comparison. The SPM[*t*] *t*‐test results indicated that the EDL SLD groups produce greater cumulative work when compared to HFD groups between WL 1 and 37 (Figure 4b. 0.12–4.40 s; *P* < 0.001).

For recovery of maximal power output relative to pre‐fatigue maximum, assessed every 10 min for 30 min post‐fatigue, in both the EDL and soleus, there was a significant main effect of time (Figure 4c, f; *P* < 0.020, η_p_^2^ = 0.656), with significant recovery of muscle power output over the 30 min. No time × HFD (*P* > 0.203, η_p_^2^ < 0.052), time × VD (*P* > 0.202, η_p_^2^ < 0.052) or time × HFD × VD interactions were observed (*P* > 0.370, η_p_^2^ < 0.028). There was no main effect of HFD (*P* = 0.228, η_p_^2^ = 0.047 for the EDL; *P* = 0.060, η_p_^2^ = 0.110 for the soleus), VD (*P* = 0.790, η_p_^2^ = 0.002 for the EDL; *P* = 0.078, η_p_^2^ = 0.097 for the soleus) or HFD × VD interaction (*P* > 0.542, η_p_^2^ < 0.012) observed.

**FIGURE 5:**
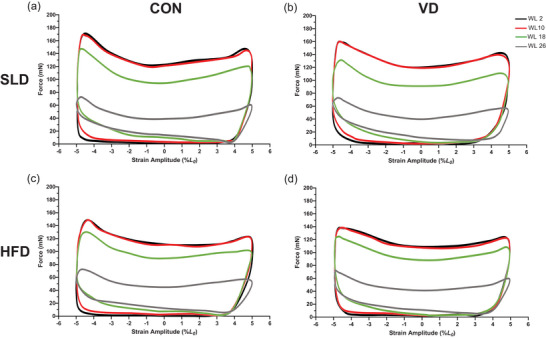
Effects of 12 weeks’ high‐fat diet and vitamin D (a, SLD; b, SLD VD; c, HFD; d, HFD VD) on average work loop shapes during muscle fatigue at 10 Hz cycle frequency for mouse EDL. Figures plotted as force against strain (%*L*_0_). Work loops 2, 10, 18 and 26 of the fatigue protocols are shown for each group. Work loops are performed in the anti‐clockwise direction, with the work loop starting at *L*_0_; *n =* 10 for SLD VD, *n =* 9 for HFD, *n =* 8 for SLD and HFD VD.

**FIGURE 6:**
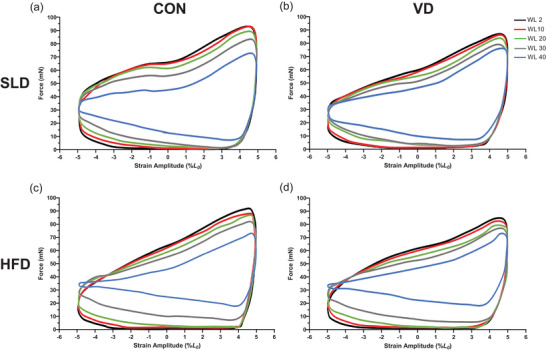
Effects of effect of 12 weeks’ high‐fat diet and vitamin D (a, SLD; b, SLD VD; c, HFD; d, HFD VD) on average work loop shapes during muscle fatigue at 5 Hz cycle frequency for mouse soleus. Figures plotted as force against strain (%*L*_0_). Work loops 2, 10, 20, 30 and 40 of the fatigue protocols are shown for each group. Work loops are performed in the anti‐clockwise direction, with the work loop starting at *L*_0_; *n =* 10 for SLD VD; *n =* 9 for HFD VD; *n =* 8 for SLD and HFD.

Figures 5 and 6 illustrate a typical WL shape during a standardized time point for mouse soleus and EDL in each respective group. The area within the WL represents the net work done during the length change cycle. The area within the typical EDL WL traces (Figure 5) is initially smaller in HFD groups (Figure 5c, d) compared to SLD groups (Figure 5a, b), but the shape of the loop is consistent across groups. The reduction of the area within the loop, and subsequent change in WL shape exhibited during the fatigue protocol appears uniform irrespective of diet or treatment. However, typical soleus WL shapes (Figure 6) demonstrate that at the later stages of the fatigue protocol (WL 30 and onward) there is greater force production during muscle re‐lengthening in HFD groups (Figure 6c, d) when compared to SLD groups (Figure 6a, b). As a result, from WL 30 there is a marked reduction in the size of WL shape relative to the initial WL in HFD groups.

## 4 DISCUSSION

The present study is the first to evaluate the effects of high dose VD on force, power and fatigue resistance of isolated mouse fast and slow twitch muscle, in both standard and high‐fat diets. Using a methodological approach, which incorporated multiple modes of contractility, it was established that a HFD evokes muscle and contractile mode specific reductions in the contractile performance of isolated skeletal muscle. In the soleus, there was a HFD‐induced decline in PO normalized to body mass and cumulative work production during fatigue. However, in the EDL there was a reduction in isometric stress, absolute PO, PO normalized to muscle mass (muscle quality) and body mass, and cumulative work. Irrespective of diet, mode of contractility or muscle phenotype, a high dose of vitamin D_3_ (VD) over a 12‐week period had little effect on contractile function of isolated soleus or EDL.

### 4.1 High‐fat diet effects on maximal muscle force and power

With respect to the effect of a HFD, the results of the present study add to the evidence demonstrating detrimental effects of HFD consumption on normalized muscle force and power in a contractile mode and muscle specific manner. However, the results extend the understanding of HFD effects by indicating for the first time that a HFD can evoke reductions in absolute power and cumulative work production of fast twitch skeletal muscle.

The isometric function (absolute or stress) of the EDL is commonly diminished in response to longer periods (>12 weeks) of HFD consumption (Eshima et al., 2020, 2017; Tallis et al., 2017), although this is not always the case (Bott et al., 2017; Hurst et al., 2019). The HFD‐induced reduction in isolated EDL stress reported in our results supports the idea that HFD can reduce the intrinsic force producing capacity of skeletal muscle. Furthermore, EDL absolute and normalized PO, and muscle quality, were diminished in the HFD groups. A HFD‐induced reduction in EDL muscle quality is supported by previous work (HFD 16 and 12 weeks, respectively; Tallis et al., 2017; Hurst et al., 2019), but the reduction in absolute EDL power output is a unique and novel insight into the impact of HFD consumption on fast twitch muscle performance. Discrepancies in findings of absolute power may be attributed to increased magnitude of intramuscular lipids occurring earlier in the feeding duration in the current study when compared to previous work. Elevated intramuscular lipids occurred in the EDL after ∼12 weeks of a fixed HFD (Eshima et al., 2017), but not until ∼16 weeks in a high‐fat forage diet (Messa et al., 2020), such as that utilized in previous work using the WL technique (Hurst et al., 2019; Tallis et al., 2017). Previous work has established that intramuscular lipids, evoked via glycerol injection, result in a reduction in the contractile capacity of whole isolated skeletal muscle (Biltz et al., 2020). Furthermore, intramuscular lipids are also linked with a reduction in the process of myogenesis (Akhmedov & Berdeaux, 2013), another key factor regulating contractile performance. HFD effects on contractile performance appear more pronounced in fast twitch muscle, attributed to fast glycolytic fibres having a reduced capacity to metabolize elevated lipid levels within muscle, when compared to muscles comprising slow oxidative fibres (Hurst et al., 2019; Tallis et al., 2017). The present data support the idea that HFD consumption effects on skeletal muscle may be contractile mode and muscle specific (Ciapaite et al., 2015; Hill et al., 2019; Tallis et al., 2018). Obesity‐induced reductions in SkM contractile performance could promote a negative obesity cycle whereby impaired SkM function contributes to and exacerbates excessive whole body lipid accumulation and disease.

Peak isometric force and force normalized to CSA (stress) of the soleus was not affected by a HFD, in concordance with the literature using similar feeding durations (Hurst et al., 2019). During longer feeding durations (16 weeks) in female CD‐1 mice, Tallis et al. (2017) identified greater absolute force of soleus from HFD animals. The magnitude of increase in body mass was originally suggested as the primary reason for an increase in absolute force observed over longer feeding durations, increasing demand on postural muscles to support and stabilize an elevated mass (Tallis et al., 2017). However, based on the present data, the concomitant effects of magnitude and duration of HFD, and duration of mechanical loading, may account for previously observed greater absolute force, as the final body masses for the HFD group from the present data (48.8 ± 1.4 g) were comparable to studies utilising longer feeding durations (16 weeks: 52.7 ± 2.3 g). Greater absolute force of soleus from HFD mice presented by Tallis et al. (2017) could be a result of the greater soleus muscle mass of HFD‐treated mice compared to controls, which was not found in the present study. Absolute soleus PO and PO normalized to muscle mass (muscle quality) were unaffected by a HFD, supporting previous observations utilising feeding durations of 12–16 weeks (Hurst et al., 2019; Tallis et al., 2017). Thus, despite elevated loading on postural muscles, no muscular adaptations occur to increase absolute power, which is further compounded by a substantial increase in body mass, supported by these data identifying a reduction in PO normalized to body mass. Therefore, despite the preservation of muscle quality in HFD groups, the magnitude of change in body mass evoked by a HFD leads to a reduction in normalized PO, where in vivo this would result in reductions in the ability to be physically active and to perform activities of daily living.

### 4.2 High‐fat diet effects on fatigue resistance

With respect to percentage decline in fatigue, these data suggest that a HFD may not influence the rate at which muscle loses power relative to maximum PO during fatiguing contractions. However, there is little consensus regarding HFD effects on isolated skeletal muscle fatigue during dynamic conditions in young adult female CD‐1 mice, with no change or a reduction in the ability to maintain PO relative to maximum being reported in both the soleus and EDL (Tallis et al., 2017; Hurst et al., 2019). Differences in fatigue response may be attributed to the use of submaximal stimulation frequency during fatigue, opposed to that which evokes maximal isometric force. Submaximal stimulation parameters provide a better representation of in vivo fatigue mechanics (Shelley et al., 2022); previous reductions in rate of fatigue may have been exacerbated by mechanical fatigue. Furthermore, previous work has only considered HFD effects on percentage decline during fatigue from a standardized point, typically maximum PO or force. Whilst this provides an insight into the performance of the tissue during fatigue, cumulative work is also an important factor when considering in vivo function, as the amount of work, in addition to the rate of fatigue, will play a key part in exercise capacity and in the ability to complete activities of daily living which require repetitive contractions.

Whilst the rate of fatigue was unaffected by HFD, total cumulative work was reduced in the HFD soleus and EDL. The reduction in cumulative work is not a result of the submaximal stimulation frequency leading to a greater reduction in PO in the HFD group, as the magnitude of change in maximal PO through a reduction in stimulation frequency did not differ between diet and treatment groups. A reduction in cumulative work in HFD EDL is likely a consequence of the reduction in acute PO, supported by work loop shapes and SPM analysis indicating greater cumulative work production during initial WL contractions. Therefore, despite HFD EDL fatiguing at a similar rate as SLD EDL, work per contraction is lower culminating in lower work production throughout the time course of fatigue. The same justification for a reduction in soleus cumulative work is unlikely given there were no differences in acute PO, suggesting other mechanisms account for a reduced capacity to perform work. It is possible that a HFD‐induced reduction in the function of sarco(endo)plasmic reticulum Ca^2+^‐ATPase (SERCA) (Funai et al., 2013), which is responsible for the movement of Ca^2+^ from the cytoplasm back into the sarcoplasmic reticulum, impairs calcium reuptake during fatiguing contractions of the soleus. Consecutive fatiguing contractions have been shown to increase relaxation time in some muscles (Allen et al., 2008); if this response is exacerbated by a HFD, it would likely cause a reduction in cumulative work as the magnitude of negative work would increase per WL cycle, that is, the muscle is active during lengthening, thus decreasing net work to a greater extent at each subsequent contraction. This is supported by the soleus WL shapes (Figure 6) – as the fatigue protocol progresses, HFD‐treated muscle had a larger negative work component during re‐lengthening, indicative of increased relaxation time, reducing work production during each cycle. However, independent of changes in SERCA, HFD‐induced reductions in the efficacy of actin–myosin cross‐bridge cycling (Ciapaite et al., 2015; Schilder et al., 2011) may play an important role in a reduction in cumulative work in both the soleus and EDL, and in the case of the soleus, without influencing acute power production.

### 4.3 Vitamin D effects on maximal force, power and fatigue resistance

The administration of high doses of VD has been shown to beneficially alter mechanisms associated with a HFD‐induced decline in skeletal muscle contractility (Benetti et al., 2018; Manna et al., 2017; Marcotorchino et al., 2014). However, the dose of VD used in the present study did not elicit any changes in whole body or muscle morphology, or any of the contractile parameters assessed, in either SLD or HFD. There are limited comparisons to be made regarding the potential for VD to alleviate obesity‐induced declines in contractile performance, and in fact, only one previous study has directly considered this (Kim et al., 2020). This previous research study reported that a high dose of VD attenuated the reduction of in vivo grip strength and sensorimotor function in 24 week old, male, p62‐deficient mice (genetic obesity model) (Kim et al., 2020). However, it is difficult to make a direct comparison with the present data, given the differences in the methodological approach, such as different model of obesity (dietary vs. genetic), gender and age of mouse, different assessment of contractile function (in vivo vs. isolated muscle) and different quantity, duration, and mode of administration of VD (75 IU every 3 days for 10 weeks via oral gavage), which are all factors that could influence the effect of VD on contractile function.

Previous research has established high dose VD for 4 weeks can evoke improvements in isolated skeletal muscle contractility when compared to control VD sufficient mice (Debruin et al., 2019; Hayes et al., 2019). Much like previous work which assessed VD supplementation on isometric properties of isolated muscle utilising the same dose (20,000 IU/kg^−1^ feed) in SLD C57/BL6 mice, no large changes in calcium handling were observed (Debruin et al., 2019, 2020; Hayes et al., 2019) as inferred by lack of difference in measurers of THPT and LSHR. Our data show that high dose VD had little effect on isometric force, WL PO or fatigue resistance of either the soleus or the EDL, in either dietary group. Previous observations support limited VD effects on EDL force output in SLD‐treated mice (Debruin et al., 2019, 2020; Hayes et al., 2019; Ray et al., 2016). However, we are the first to show minimal effects of high dose (20,000 IU/kg^−1^ feed) VD on soleus stress, as prior work indicates increased soleus stress after 4 weeks (Debruin et al., 2019; Hayes et al., 2019) but diminished after 8 weeks (Debruin et al., 2020). It could be that excess VD supplementation adversely affects muscle function, as has been reported in both human and animal studies, where large single bolus doses have been associated with impaired isolated muscle contractile performance (Hayes et al., 2019) and increased risk of falls in the elderly (Sanders et al., 2010), although the dose provided in this study, whilst high, was not given in one bolus dose and nor do the data show a reduction in contractile performance irrespective of muscle or mode of contractility. Whilst speculative, based on previous evidence and the data presented in this study, there could well be an ‘inverted‐U’ relationship with duration of high dose VD supplementation and isolated skeletal muscle contractility (Bollen et al. 2022). Initially a high dose of VD may be beneficial for isolated skeletal muscle contractility until an optimal quantity of circulating VD is achieved, and from that point on there is a downward trajectory in muscle performance; we suspect our data may fit in this downward trajectory. However, more data are needed to explore the effect of dose and duration of VD on isolated muscle contractility.

Despite the findings presented in this study, high dose VD could still be beneficial for contractile performance when supplemented long term during HFD feeding. Throughout the data presented there are consistent small (*d* = 0.2–0.59) and moderate (*d* = 0.6–1.19) effect sizes suggesting improved contractile performance of the EDL (absolute and normalized PO and stress) and lower fat mass and fat mass: body mass in HFD VD when compared to HFD only. Whilst these effect sizes should be interpreted with caution, they are important to note as they further contextualize that VD could improve contractile function and morphology in HFD‐treated rodents when feeding parameters are optimized, as opposed to VD having no anti‐obesogenic properties. The response to VD appears dose/duration dependent, whereby excess VD may cause VD dysregulation and result in deleterious (Debruin et al., 2020), or in the case of these data, no significant effects on contractile performance. The reduction (∼50%) in isometric stress observed after 8 weeks’ high dose VD was attenuated when supplemented with exercise and even enhanced fatigue resistance compared to exercise only (Debruin et al., 2020). Debruin et al. (2020) suggest that high dose VD alone results in vitamin D dysregulation, through alterations in the activity of key metabolic enzymes involved in vitamin D metabolism (e.g., 1α‐hydroxylase and 24α‐hydroxylase). However, exercise‐induced enhancement of mitochondrial function appeared to abate the increase in metabolic stress evoked by high dose VD, ultimately promoting improvements in contractile function (Debruin et al., 2020). Therefore, it may be that high dose VD is beneficial in attenuating a HFD‐induced decline in muscle contractility when combined with an added stimulus which evokes beneficial alterations in mitochondria, be that exercise or a combination with other nutritional strategies.

The effects of surplus VD on isolated muscle function appear dependent upon a number of factors such as dose, duration, sex and physical activity. There is evidence demonstrating that chronic supplementation of high dose VD can improve isolated muscle performance (Debruin et al., 2019; Hayes et al., 2019) and evoke mechanisms which directly oppose HFD‐induced declines in muscle function (Benetti et al., 2018; Manna et al., 2017; Marcotorchino et al., 2014). Given the available evidence, coupled with the need to explore alternative therapeutic strategies to alleviate the adverse effects of obesity, supplementation of VD warrants further investigation; understanding and identification of suitable dosing strategies could contribute to a reduction in the adverse effects of obesity on overall health and specifically skeletal muscle health (Tallis et al., 2021), which are current public health priorities.

### 4.4 Limitations and future direction

One limitation of this study is that the exact levels of VD achieved are unknown, as serum 25(OH)D concentration in blood plasma was not measured. Given that previous studies have observed increased quantities of VD in adipose tissue of both obese human and rodents, when compared to lean controls (Szymczak‐Pajor et al., 2022) it is possible that serum 25(OH)D concentration in blood plasma could be different between VD‐treated groups despite receiving an identical dose. However, given that we observed no changes in SkM contractility in either VD‐treated groups compared to their respective control, we suspect that VD stored in adipose tissue alone would not fully explain why no changes in tissue mechanics were observed. Furthermore, previous research shows that an identical dose and duration (20,000 IU/kg diet for 12 weeks) elicits ∼4‐fold increase in serum 25(OH)D concentration when compared to a control dose (1000 IU/kg diet) in C57 mice (Rowling et al., 2007). Thus, we are confident that VD groups would have substantially greater quantities of circulating VD compared to controls. Another limitation of the present study is that dietary consumption was not monitored as animals were socially housed as a means to avoid any unnecessary stress upon the animals which can occur through individual housing (Liu et al., 2020). Thus, exact quantity of HFD or VD consumed could not be quantified. As there were no histological investigations on the tissue, there is still speculation as to the underpinning mechanisms which evoked HFD‐induced reductions in SkM function, or whether mechanistic changes occurred in VD‐treated animals despite no change in contractile function. Future work may wish to consider the role of intramuscular lipid content on HFD‐induced reductions in muscle quality. In the present study absolute power was normalized to whole muscle mass. However, as skeletal lipid content can be two‐fold greater in HFD‐treated rodents (Messa et al., 2020), if PO were normalized to lean tissue mass, reductions in muscle quality could be less pronounced. Although, given the magnitude of a decline in muscle quality observed, a decline in muscle quality would still be expected given that SkM lipid content makes up a small portion of whole muscle mass irrespective of dietary consumption (Machann et al., 2003).

Whilst the present study provides a valuable insight into the effects of VD on isolated muscle mechanics, these results are only generalizable to the specific dose and duration used, in female mice. One plausible explanation for differences between our data and previous work may well be sex specific responses, given there may be sex specific determinants in the modulation of 25(OH)D levels (Dupuis et al., 2021; Jungert & Neuhäuser‐Berthold, 2015) – there is limited available evidence for how this affects the contractile response. Overall previous studies provide evidence that VD supplementation can, in some instances, promote improvement in mechanisms responsible for optimal skeletal muscle performance, including attenuation of mechanisms responsible for HFD‐induced declines in contractile performance such as reduced AMPK activity and decreased lipid metabolism (Benetti et al., 2018; Manna et al., 2017; Marcotorchino et al., 2014). As such, future work is needed to establish the optimal dose and duration of VD to evoke improvements in isolated skeletal muscle mechanics in both SLD‐ and HFD‐treated female and male mice.

### 4.5 Conclusion

The present study demonstrates that HFD effects are muscle and contractile mode specific. For force and power performance there are limited effects of HFD on isolated mouse soleus, but HFD evokes reductions in EDL stress and absolute and normalized PO. During fatiguing contractions, whilst percentage decline relative to maximum PO remained unchanged between groups, total cumulative work was reduced in HFD‐treated soleus and EDL, although it would appear the mechanisms responsible for this decline are not uniform between muscles. Whilst high dose VD did not attenuate any of the HFD‐induced declines in contractile performance or alter contractile performance in SLD‐treated mice in this study, there were small and moderate non‐significant effects observed between HFD VD and HFD groups to suggest refinement of feeding protocols may evoke positive effects on contractile performance and morphology. As such, future work should consider the impact of various doses and durations to determine if VD can be effective in reducing the impact of obesity of skeletal muscle function.

## AUTHOR CONTRIBUTIONS

Sharn P. Shelley, Jason Tallis and Rob S. James conceived and designed the study; Sharn P. Shelley performed data collection; Sharn P. Shelley and Jason Tallis analysed the data; Sharn P. Shelley prepared figures; Sharn P. Shelley and Jason Tallis drafted manuscript; all edited and revised the manuscript. All authors have read and approved the final version of this manuscript and agree to be accountable for all aspects of the work in ensuring that questions related to the accuracy or integrity of any part of the work are appropriately investigated and resolved. All persons designated as authors qualify for authorship, and all those who qualify for authorship are listed.

## CONFLICT OF INTEREST

The authors declare they have no competing or conflict of interest.

## FUNDING INFORMATION

No external funding was received for this work.

## Data Availability

Source data and statistical parametric mapping outputs are available in FigShare at https://doi.org/10.6084/m9.figshare.24420946.v1.
